# Effects of nitrogen input on soil bacterial community structure and soil nitrogen cycling in the rhizosphere soil of *Lycium barbarum* L.

**DOI:** 10.3389/fmicb.2022.1070817

**Published:** 2023-01-10

**Authors:** Yuekun Li, Nan Zou, Xiaojie Liang, Xuan Zhou, Shuhan Guo, Yajun Wang, Xiaoya Qin, Yehan Tian, Jin Lin

**Affiliations:** ^1^National Wolfberry Engineering Research Center, Wolfberry Science Research Institute, Ningxia Academy of Agriculture and Forestry Sciences, Yinchuan, China; ^2^Shandong Provincial Key Laboratory for Biology of Vegetable Diseases and Insect Pests, College of Plant Protection, Shandong Agricultural University, Taian, China

**Keywords:** *Lycium barbarum* L., bacterial community, nitrogen cycle, goji berry quality, rhizosphere soil

## Abstract

*Lycium barbarum* L., goji berry, is a precious traditional Chinese medicine and it is homology of medicine and food. Its growth is heavily dependent on nitrogen. The use of chemical fertilizers has significantly promoted the yield of goji berry and the development of the *L. barbarum* L. industry. However, crop plants are inefficient in the acquisition and utilization of applied nitrogen, it often leads to excessive application of nitrogen fertilizers by producers, which cause negatively impact to the environment ultimately. The exploration of an interaction model which deals with crops, chemical fertilizers, and rhizosphere microbes to improve nitrogen use efficiency, is, therefore, an important research objective to achieve sustainable development of agriculture greatly. In our study, we explored the effects of nitrogen input on soil microbial community structure, soil nitrogen cycling, and the contents of nutrients in *L. barbarum* fruits. The structure and composition of the bacterial community in the rhizosphere soil of *L. barbarum* were significantly different under different nitrogen supply conditions, and high nitrogen addition inhibited the diversity and stability of bacterial communities. Low nitrogen input stimulated the relative abundance of ammonia-oxidizing bacteria (AOB), such as *Nitrosospira*, catalyzing the first step of the ammonia oxidation process. The results of the GLMM model showed that the level of nitrogen fertilizer (urea) input, the relative abundance of AOB, the relative abundance of *Bradyrhizobium*, and their combinations had significant effects on the soil nitrogen cycling and contents of nutrients in *L. barbarum* fruits. Therefore, we believe that moderately reducing the use of urea and other nitrogen fertilizers is more conducive to improving soil nitrogen use efficiency and Goji berry fruit quality by increasing the nitrogen cycling potential of soil microorganisms.

## 1. Introduction

*Lycium barbarum* L. is a perennial deciduous shrub belonging to the *Lycium* genus of the Solanaceae family. Its fruit, goji berry, is a precious traditional Chinese medicine and also a functional food. It has the functions of tonifying the kidney, nourishing the liver, the lung, and the eyes, enhancing immunity, preventing aging, fighting tumors, etc. (Meng et al., 2019). Ningxia is the main production area of wolfberry, and the artificial cultivation of wolfberry originated in Tang and Song Dynasties (Wang et al., 2019). Among the *Lycium* species, *Lycium barbarum* L. is the only variety selected as a medicine according to the Chinese Pharmacopeia, and it is also the most influential product of geographical indication in Ningxia. The wolfberry industry, as a strategic leading industry with distinct advantages, has become a potential growth point in the economy of Ningxia in recent years.

In recent years, the importance of the rhizosphere microbiome for plant growth has been widely recognized and become a hotspot in the field of agriculture (Mendes et al., 2013). With the application of non-culture methods such as molecular biology and genomics, the number of studies on microorganisms of the rhizosphere of *Lycium* is increasing year by year (Li et al., 2022). These research articles have mainly focused on the effects of continuous cropping and years of planting on the rhizosphere microbial community diversity of *L. barbarum*, as well as the screening of growth-promoting bacteria, arbuscular mycorrhizal fungi (AMF), and other microbes (Ma et al., 2019; Na et al., 2021). It has been observed that the composition of the rhizosphere soil microbial community was greatly affected by planting years and zones (Na et al., 2016; Li et al., 2022b). The continuous cropping had more negative effects, significantly inhibiting the growth of replanted wolfberry, and the continuous cropping barrier affected the structure and diversity of rhizosphere bacterial communities and interfered with the interaction between the wolfberry plant and soil bacterial communities. Long-term application of chemical fertilizers may be one of the main factors that change fungal community structure and the interaction between replanted Chinese wolfberry roots and fungi in fields under continuous cropping (Ma et al., 2019). To solve the problem of the continuous cropping barrier of wolfberry, research on microorganisms of the rhizosphere of wolfberry was conducted by screening the growth-promoting bacteria and AMF (arbuscular mycorrhizal fungi; Chen et al., 2022; He et al., 2022; Li et al., 2022a).

Nitrogen is an essential nutrient for plant growth and basic metabolic processes. Most plants depend on available forms of nitrogen for growth, such as ammonium salts and nitrates. Soil nitrogen availability depends on different nitrogen conversion reactions, which are performed by a complex network of microorganisms with a variety of metabolic functions (Torres et al., 2016; Khan et al., 2017; Anas et al., 2020; Tiong et al., 2021). Urea is the most widely used nitrogen fertilizer in crop production in China (Anas et al., 2020). After it is applied, urea would undergo leaching, volatilization, nitrification, and denitrification, and the loss rate of nitrogen is as high as over 45% (Liang et al., 2007; Yu et al., 2010). Most microbes are still only able to perform one or more steps of the nitrogen conversion process, and therefore, nitrogen conversion requires the cooperation of a diverse microbial community. However, the long-term excessive application of nitrogen fertilizers, such as urea, will change the microbial community diversity, and thus, affect the normal nitrogen cycle in soil (Ma et al., 2007; Lv et al., 2017; Chen et al., 2019).

However, there are still many unsolved problems with mechanisms that the effects of nitrogen inputs on soil nitrogen cycling and nitrogen utilization by changing microbial community in the rhizosphere soil of plants. This study aims at unraveling the unclear mechanisms of nitrogen conversion and putting forward an exploration of improving low nitrogen fertilizer use efficiency of *L. barbarum*. Therefore, the main wolfberry cultivar “Ningqi No.7” was taken as the research material, and concentration gradients of nitrogen treatments were used to exhibit three nitrogen states of *L. barbarum* plants, including nitrogen deficiency, nitrogen normal, and nitrogen excess. By sequencing and q-PCR quantitative analysis of the rhizosphere soil microbial, the responses of nitrogen-transforming microorganisms of wolfberry rhizosphere soil to differences in nitrogen application rates were evaluated. At the same time, the changes in the metabolome of wolfberry fruits in different treatments were determined to study the changes in fruit secondary metabolites caused by different nitrogen application rates. Based on this, the structure and function of microorganisms driving the conversion of nitrogen in the rhizosphere of wolfberry were investigated.

## 2. Materials and methods

### 2.1. Site description and experimental design

The experiment with different N treatments was established in the Siying Township, Haiyuan County, Zhongwei City, NingXia, China (36°25′48″N, 106°09′00″E, ~1,428.5 m altitude), with an average annual precipitation of 367.4 mm, the mean annual temperature of 7.3°C, and the average annual sunshine hours of 2,710 h.

The N addition experiment involved four levels of N enrichment: N0 (0 g·N m^−2^ year^−1^), N1 (53.82 g·N m^−2^ year^−1^), N2 (67.62 g·N m^−2^ year^−1^), and N3 (80.73 g·N m^−2^·year^−1^), and each treatment was repeated five times (Supplementary Table S1). Among them, 67.62 g·N m^−2^·year^−1^ is the most commonly used fertilization dosage in the goji field production. The five-year-old *L. barbarum* cultivar “Ningqi No.7” was used as the experimental material. In the early growth stages of the plant (October 24, 2020, April 28, 2021, May 29, 2021, and June 29, 2021), N fertilizer was applied four times according to the designed fertilizer treatment (one base fertilizer, three topdressing fertilizers).

The rhizosphere soil samples were collected in July 29, 2021 (30 days after the last fertilization). Three to five healthy plants from experimental units were randomly selected for each treatment, and the sampled trees were labeled with site-directed markers. The rhizosphere soil samples from the 0–40 cm vertical depths attached to the root cap were collected by the 5-point sampling method. The soil volume taken at each sampling point was roughly the same. After mixing, samples were loaded into frozen storage tubes, stored in liquid nitrogen tanks, and then, they were transferred to the laboratory and stored at −80°C for sequencing analysis.

Fruit samples were collected at the same time as the rhizosphere soil samples (July 29, 2021). Fresh, ripe, and pest-free fruits were collected and placed into frozen storage tubes, stored in liquid nitrogen tanks, transported to the laboratory, and stored at-80°C for metabolome analysis.

### 2.2. Determination of soil chemical properties

The soil pH was determined using a DDSJ-319L electrode pH meter (INESA Co., Ltd., Shanghai, China) with a 1:2.5 soil/water (w/v) suspension. According to the standard test methods of the Chinese national standard, other soil physicochemical properties, such as the contents of total nitrogen (TN, NY/T1121.24–2012), total phosphorus (TP, NY/T 88–1988), total potassium (TK, NY/T 87–1988), available nitrogen (AN, LY/T1228–2015), available phosphorus (AP, NY/T1121.7–2014), nitrate nitrogen and ammonium nitrogen (NO_3_^−^ and NH_4_^+^, respectively, GB/T 32737–2016), available potassium (AK, NY889–2004), and soil organic matter (OM, NY/T1121.6–2006) and electrical conductivity (EC, HJ 802–2016) were evaluated (Tian et al., 2021). The activities of β-1.4-N-acetylglucosaminidase (NAG), urease (UR), and Leucine aminopeptidase (LAP) were determined using various soil enzyme activity test kits provided by the manufacturer (Solarbio Biotechnology, Beijing, China).

### 2.3. DNA extraction, PCR amplification, and sequencing

The genomic DNA of each rhizosphere soil sample was extracted using the Omega Soil DNA Kit (Omega Bio-tek Inc., Microorganisms 2021, 10, 1644 4 of 28 Norcross, GA, United States) and quantified using the Nanodrop 2000 spectrophotometer (Nanodrop Technologies, LLC, Wilmington, DE, United States). The V3-V4 region of the 16S rRNA gene was amplified by PCR using the primer sets 338F (ACTCCTACGGGAGGCAGCAGCAG) and 806R (GGACTACHVGGGTWTCTAAT). PCR products from each sample were mixed and recovered from the 2% agarose gel. The AxyPrep DNA Gel Extraction Kit (Axygen Biosciences, UnionCity, CA, United States) was used to purify the recovered products, which were then detected by 2% agarose gel electrophoresis and quantified using a Quantus™ Fluorometer (Promega, United States).

### 2.4. Sequence and statistical analyses

The raw sequencing data were first subjected to quality control as described by Schmieder and Edwards (2011). Microbiome bioinformatics were performed with QIIME 22019.4 (Bolyen et al., 2019) with slight modification according to the official tutorials.^1^ Sequences were then quality filtered, denoised, merged and chimera removed using the DADA2 plugin (Callahan et al., 2016). The filtered sequences were then clustered into operational taxonomic units (OTUs) at a 97% identity threshold. The taxonomy of representative OTUs was annotated according to their RDP Classifier, and BLAST queries were performed against the Silva and NCBI databases (Quast et al., 2013). OTUs with an RDP classification threshold that was below 0.8 or with <90% identity and coverage were denoted as unclassified. All OTUs identified as plastid, and mitochondrial DNA were removed.

Mothur 1.30.1 was used for rarefaction analysis, and the vegan 2.4.2 package was used to calculate the alpha-diversity indices, including Chao1, Observed species, Shannon, Simpson, Pielou’s evenness and Good’s coverage. A one-way analysis of variance (ANOVA) was used to determine the significant differences among the three nitrogen levels and four growth stages. Histograms were generated using the statistical software package R to illustrate the community structure. Principal coordinates analysis (PCoA) was performed to evaluate the beta diversity of microbial communities. The analysis of similarities (ANOSIM) and permutational multivariate analysis of variance (PERMANOVA) were performed to evaluate the similarity of the microbial community structure, as well as the effects of different growth cycles and spatial compartments on community composition (McArdle and Anderson, 2001; Warton et al., 2012). To further explain the differences between the compartments and growth stages, compartment-specific and growth stage-specific biomarkers were identified, respectively, by using the linear discriminant analysis effect size (LEfSe) with linear discriminant analysis (LDA) > 3.

### 2.5. Sequencing analysis of the fruit metabolome

Sample preparation: The *L. barbarum* fruit samples were vacuum freeze-dried (ScientZ-100F) and ground to powder using a grinder at 30 Hz for 1.5 min (MM 400, Retsch). Then, 100 mg of powder was dissolved in 1.2 ml of the 70% methanol extract, vortex-mixed six times, each time for 30 s every 30 min. The samples were then placed in the refrigerator at 4°C overnight. After centrifugation at 12,000 rpm for 10 min, the supernatant was taken and filtered with a 0.22 μm microporous membrane before analysis with a device.

The sample extracts were analyzed using a UPLC-ESI-MS/MS system (UPLC, SHIMADZU Nexera X2 system; MS, Applied Biosystems 4500 Q TRAP) equipped with the Agilent SB-C18 column (1.8 μm, 2.1 mm × 100 mm). The column temperature and the injection volume were set to 40°C and 4 μl, respectively. The mobile phase consisted of the solvent (mobile phase) A (pure water with 0.1% formic acid) and solvent (mobile phase) B (acetonitrile with 0.1% formic acid). The gradient program was described as follows: 0–9 min, 5–95% B; 9–10 min, 95% B; 10–11 min, 95–5% B; 11–14 min, 5% B. The MS System was equipped with an Electrospray Ionization (ESI; Turbo Ion-Spray) interface, and the operating parameters were as follows: an ion source, turbo spray; source temperature of 550°C; ion spray voltage (IS) of 5,500 V (positive ion mode)/−4,500 V (negative ion mode); and ion source gas I (GSI), gas II (GSII), and curtain gas (CUR), which were set at 50, 60, and 25.0 psi, respectively; the collision gas (CAD) was set high.

After loading, the obtained LC-MS raw data were imported into the software of Progenesis QI designed for metabolomics analysis (Waters Corporation, Milford, MA, United States) for baseline filtering, peak identification, integration, alignment, and retention-time correction. Finally, the plot was constructed by using a retention time, the mass-to-charge ratio, and peak intensity data matrix to remove the missing values from the data that exist in more than 20% of samples (80% rule), which would include at least – group of samples of China-Africa with the zero value appearing in more than 80% of the variable, to fill the gap of the minimum missing values on the original matrix to reduce the instability in sample preparation and the error of a measuring instrument. The response intensity of spectrum peaks of the sample was normalized by the summation normalization method, and then the normalized data matrix was obtained. At the same time, variables with a relative standard deviation (RSD) > 30% of QC samples were deleted. The preprocessed data were uploaded to the sequencing company’s cloud-based platform for analysis. The PCA and orthogonal partial least squares discriminant analysis (OPLS-DA) were performed using RoPLS (Version1.6.2) R package, and the stability of the model was evaluated by seven-cycle cross-validation. The selection of significantly different metabolites was based on the variable weight value (VIP) obtained from the OPLS-DA model and the *p*-value derived from the Student’s *t*-test (Chen et al., 2013). The different metabolites of different treatment groups were screened out.

### 2.6. Quantitative PCR analysis

The abundances of AOA, AOB, and NIFH were quantified by real-time PCR using the primer pairs *Arch-amoA*F/*Arch-amoA*R, *amoA1*F/*amoA2*R, and *nifH*-F/*nifH*-R (Supplementary Table S2), respectively. To standardize the quantification of the *AOA*, *AOB*, and *NIFH* genes, they were PCR-amplified from extracted DNA with the primer pairs *Arch-amoA*F/*Arch-amoA*R, *amoA1*F/*amoA2*R, and *nifH*-F/*nifH*-R, respectively. The standard curves were generated using 10-fold serial dilutions of plasmids containing the *AOA*, *AOB*, or *NIFH* target gene inserts. Each PCR reaction mixture contained 10 μl of SYBR Premix Ex Taq TM (Takara, Dalian, China), 0.4 μl of each of the 10-μM forward and reverse primers, 7.2 μl of sterilized MilliQ water, and 2 μl of the standard or extracted DNA from soil. The PCR was performed using the Light Cycler® 96 instrument (Roche Applied Science). First, the copy numbers for AOA, AOB, or NIFH genes were calculated using a regression equation for converting the cycle threshold (Ct) value to the known number of copies in the standards. Then, the copy numbers of AOA, AOB, or NIFH genes were converted into copies per gram of dry soil according to the paper published by Gao et al. (2013).

### 2.7. Generalized linear mixed model (GLMM) analysis

To further investigate the effects of bacterial structural variables on the nutrient contents of wolfberry fruits and soil nitrogen cycle, we analyzed the data by regression using the generalized linear mixed model (GLMM). The GLMM was used with the “glm” function in “lme4” package under R version 3.6.1. with 17 dependent variables [UL, bacterial alpha-diversity (Shannon, Simpson, ACE, Chao, and Coverage), and the abundance of bacteria involved in the nitrogen cycle (NIFH, AOA, AOB, *bacillus*, *nitrospira*, *pseudomonas*, *nitrosospira*, *bradyrhizobium*, *nitrosomonas*, *nitrolancea*, and *nitrococcus*)] and 15 independent variables [the nutrient contents of wolfberry fruits (AA, Fla., Sac, Alk, and Vit) and soil physical and chemical properties (pH, EC, OM, TN, TP, TK, NO_3_^−^, NH_4_^+^, AK, and AP)]. We established different types of the model and considered different bacterial structural variables as fixed effects and sample types as random variables in the model. The rate of convergence of the algorithm was also considered, and AIC (Akaike information criterion) was used as the evaluation index of the model. We screened and removed irrelevant factors step by step to obtain the best-fitting model.

### 2.8. Statistical analysis

The results of physiological analyses were presented as mean ± standard deviation of at least three biological replicates. Statistical analysis was performed using SPSS 22.0 software. One-way analysis of variance (ANOVA) along with the Student–Newman–Keuls test were performed to determine statistically significant differences between the means of groups.

## 3. Results

### 3.1. Soil chemical properties

**Figure 1:**
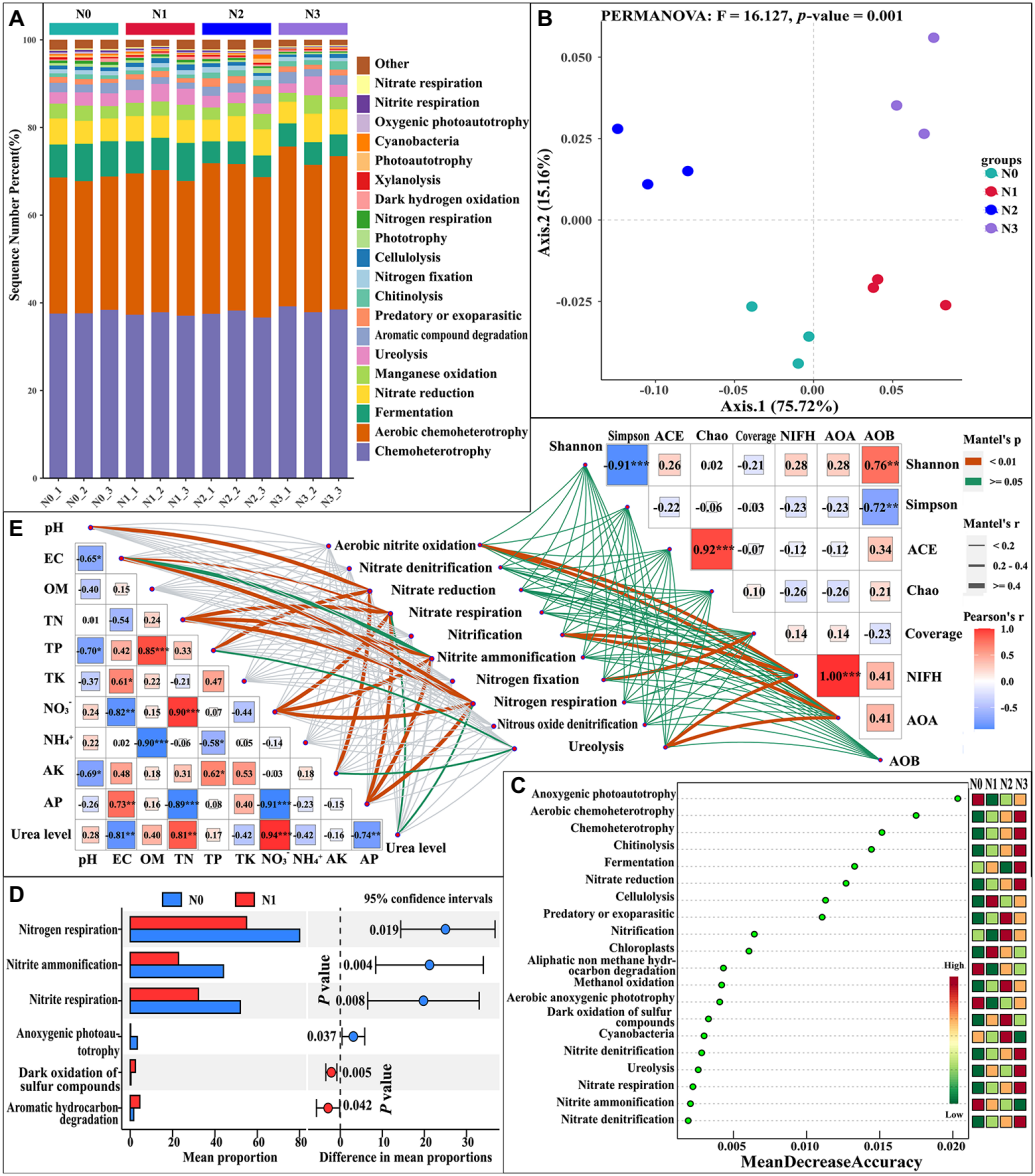
Influences of experimental N addition on microbial function and nitrogen metabolism function.

Changes in soil physicochemical properties and enzyme activities after N addition were analyzed. Although there were no significant differences in pH and total potassium (TK) content among the groups N0, N1, N2, and N3, other soil physicochemical properties and activities of soil enzymes showed significant differences (Table 1). Compared with N0 and the contents of total nitrogen (TN), nitrate nitrogen (NO_3_^−^) increased in N1, N2, and N3, the contents of ammonium nitrogen (NH_4_^+^) decreased in N1, N2, and N3. The changing trend of electrical conductivity (EC) and available phosphorus (AP) showed significantly negative correlations with the amount of added urea (*p* < 0.01). However, the changing trend of pH, and contents of total nitrogen (TN), nitrate nitrogen (NO_3_^−^) showed significantly positive correlations with the amount of added urea (*p* < 0.01; Figure 1E). Meanwhile, the activities of soil enzymes urease (UR) and β-1.4-N-acetylglucosaminidase (NAG) had negative correlations with the amount of urea added, while the activities of leucine aminopeptidase (LAP) showed positive correlations with the increased amount of urea (*p* > 0.05; Supplementary Table S3). Moreover, the activity of UR showed a significant positive correlation with the content of TN (*p* < 0.05).

**Table 1:** Response of soil chemical properties and soil enzyme activity to experimental N addition.

Treatment	N0	N1	N2	N3
pH	7.94 ± 0.03ab	7.95 ± 0.03ab	7.92 ± 0.02b	7.99 ± 0.01a
EC (mS/cm)	1.65 ± 0.02a	1.41 ± 0.04b	1.45 ± 0.01b	0.96 ± 0.02c
OM (g/kg)	7.16 ± 0.23d	14.33 ± 0.06a	13.53 ± 0.21b	7.97 ± 0.04c
TN (g/kg)	0.53 ± 0.01c	0.62 ± 0.02b	0.96 ± 0.02a	0.87 ± 0.07ab
TP (mg/kg)	0.57 ± 0.03c	0.69 ± 0.03b	0.80 ± 0.02a	0.51 ± 0.03d
TK (mg/kg)	19.10 ± 0.66a	18.90 ± 0.36a	19.07 ± 0.23a	18.40 ± 0.17a
NO_3_^−^ (g/kg)	5.66 ± 0.30d	8.09 ± 0.30c	10.98 ± 0.56b	12.28 ± 0.51a
NH_4_^+^ (g/kg)	2.45 ± 0.12a	1.77 ± 0.07c	2.07 ± 0.06b	2.19 ± 0.02b
AK (mg/kg)	176.67 ± 5.77b	163.33 ± 5.77c	190.00 ± 0.00a	163.33 ± 5.77c
AP (mg/kg)	51.64 ± 0.48b	54.43 ± 0.78a	26.94 ± 0.33c	18.90 ± 0.24d
NAG (nmol/g/h)	4.64 ± 0.37b	3.46 ± 0.12c	5.62 ± 0.42a	3.68 ± 0.35c
LAP (nmol/g/h)	22.49 ± 2.59a	23.35 ± 1.08a	25.42 ± 0.88a	22.54 ± 1.41a
UR (μg/g/h)	33.39 ± 4.00c	51.15 ± 2.27a	46.59 ± 2.66b	35.73 ± 2.88c

EC, Electrical conductivity; OM, Organic matter; TN, Total nitrogen; TP, Total phosphorus; TK, Total potassium; NO_3_^−^, Nitrate nitrogen; NH_4_^+^, Ammonium nitrogen; AK, Available potassium; AP, Available phosphorus; NAG, β-1.4-N-acetylglucosaminidase; LAP, Leucine aminopeptidase; UR, Urease. Different letters in the same column indicate significant statistical difference at *p* < 0.05 level determined using the Student-Newman-Keuls test, the below is same.

### 3.2. The contents of metabolites in *Lycium barbarum* fruits

**Figure 2:**
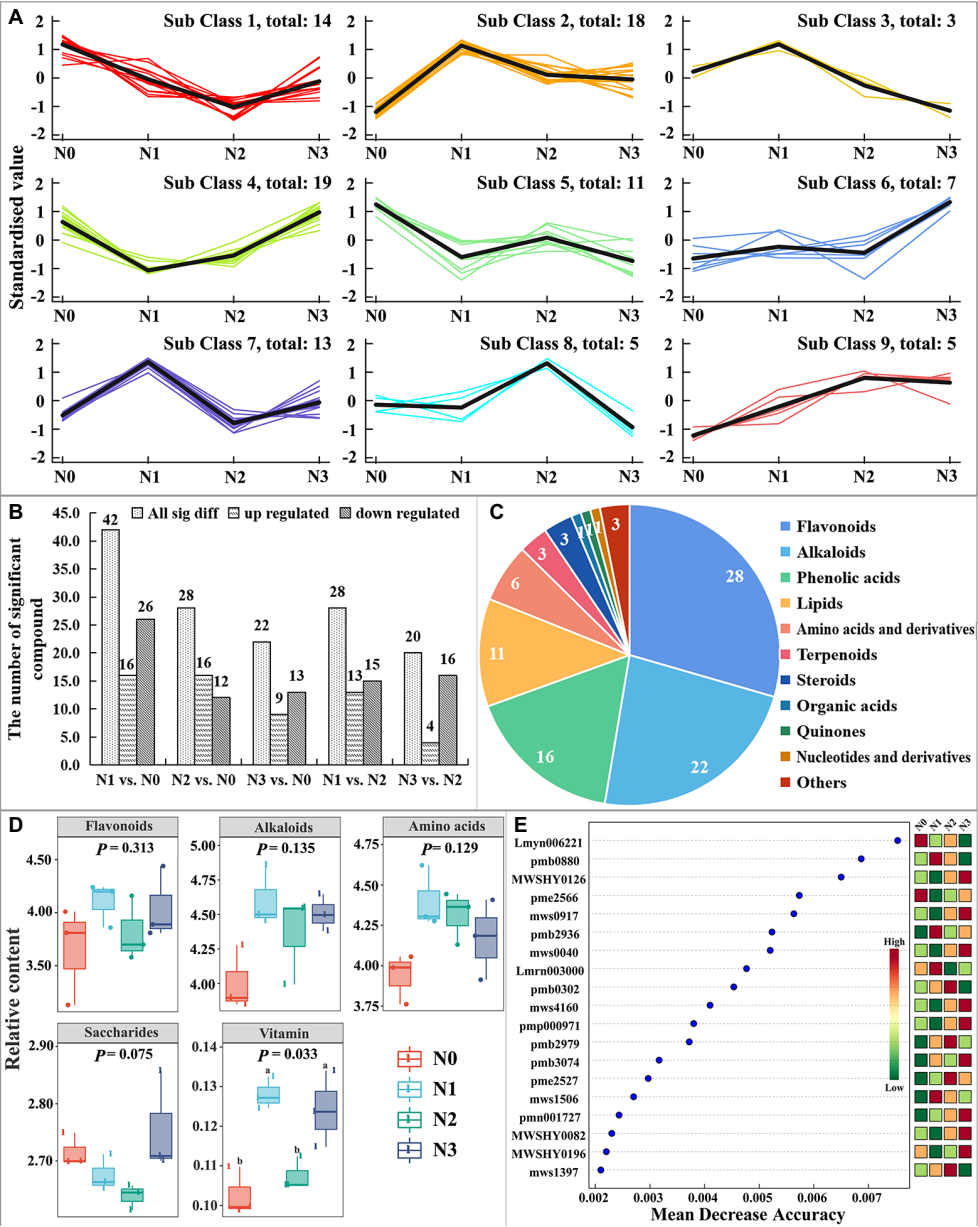
Response of the levels of fruit metabolites of *Lycium chinensis* to experimental N addition based on widely targeted metabolomics analysis.

The total ion chromatogram (TIC) of the quality control (QC) sample (a mixture of all the samples investigated) and a multi-peak detection plot of chemicals in the MRM mode of the same sample are illustrated in Supplementary Figure S1. The peaks of different colors represent the presence of different components in the sample. In the present study, a total of 1,029 metabolites were identified in the fresh *L. barbarum* samples, which were divided into nine classes based on the change in each treatment (Figure 2A). There were 42, 28, 22, 28, and 20 significantly different metabolites in groups N1: N0, N2: N0, N3: N0, N1: N2, and N3: N2, respectively (Figure 2B), including 28 flavonoids, 22 alkaloids, 16 phenolic acids, 11 lipids, 6 amino acids and derivatives, 3 terpenoids, 3 steroids, 1 organic acid, 1 quinone, 1 nucleotide and derivative, and 3 other chemicals (Figure 2C). Among them (Figure 2D), the contents of flavonoids, alkaloids, amino acids, and saccharides in control fruits (N0) had no significant differences with those of N treatment samples (N1, N2, and N3), among which, all except the content of saccharides showed an increase in N treatment samples when compared to N0. The contents of vitamins showed significant increases in N1 and N3 compared to N0 and N2, and no difference was observed between N0 and N2. The contents of flavonoids, alkaloids, and vitamins in tested samples had the same pattern of increasing first, then decreasing, and then again increasing with the increase in the amount of added urea fertilizer. To screen the potential metabolite markers under the influence of different nitrogen supply levels, the random forest analysis of the relative data of the contents of metabolites in *L. barbarum* was conducted under four nitrogen supply levels. Based on the result of LEfSe, 19 most important metabolites were selected, 8 belonged to flavonoids (Figure 2E). This result indicates that flavonoids, such as MWSHY0126 (kaempferide), mws0917 (3, 7-Di-O-methylquercetin), mws0040 (chrysin), mws4160 (wogonin), MWSHY0082 (galangin), pmb2979 (hesperetin-7-O-(6-malonyl glucoside)), MWSHY0196 (genkwanin), and mws1397 (epicatechin gallate), had the greatest impacts on the accuracy of the random forest classifier and thus can be used as metabolite markers of *L. barbarum* fruit for soil nitrogen levels (Figure 2E).

### 3.3. The microbial diversity in the rhizosphere soil of *Lycium barbarum*

#### 3.3.1. Effects of N supply levels on the α-diversity of soil bacterial community

**Figure 3:**
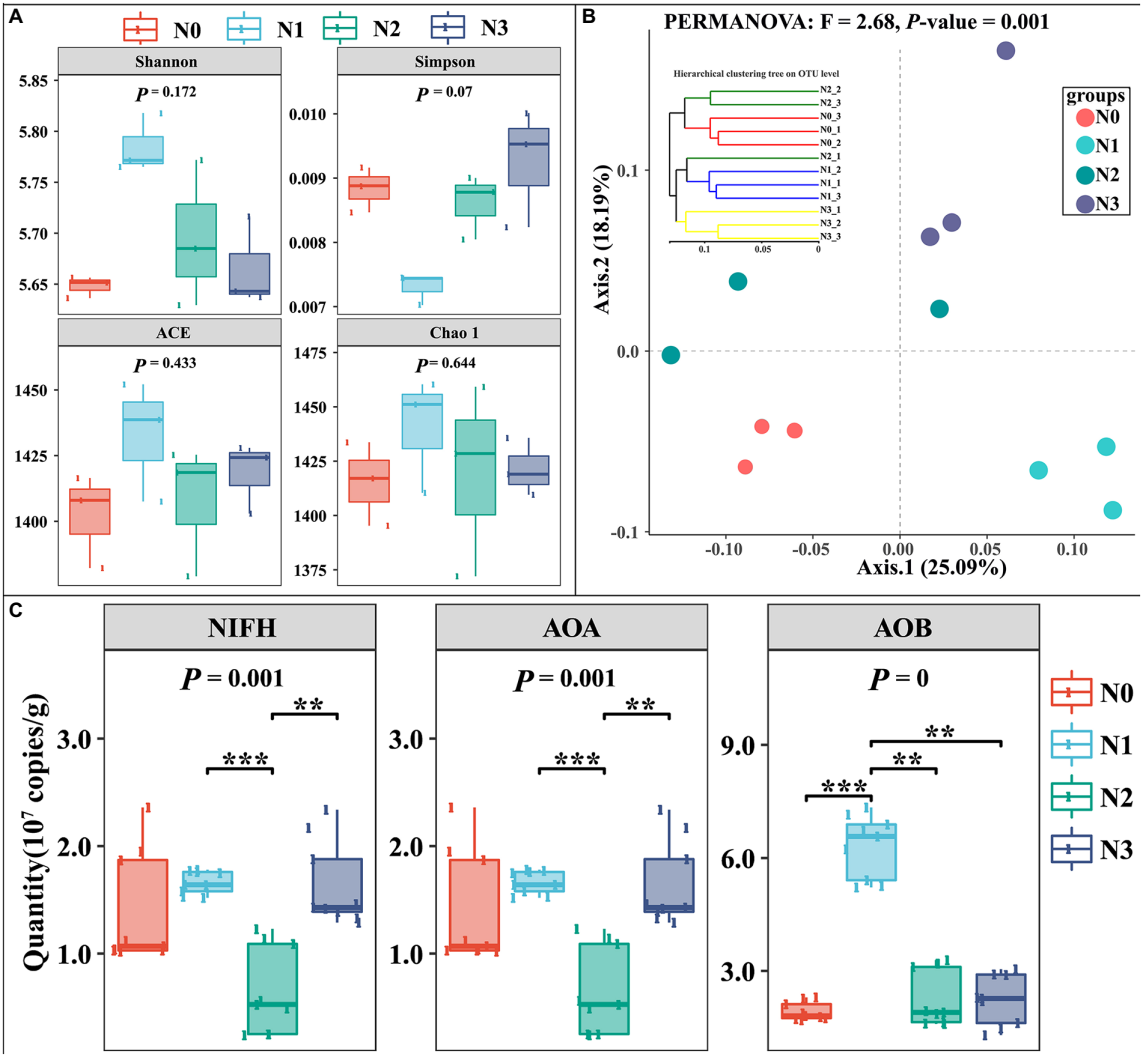
Influences of experimental N addition on microbial diversity index and community composition.

The α-diversity of the soil bacterial community was represented by the indices reflecting community richness (ACE and Chao1) and the indices reflecting community diversity (Shannon and Simpson). The α-diversity indices in control samples (N0) had no significant differences from those in N treatment samples (N1, N2, and N3; Figure 3A). But, Shannon, ACE, and Chao1 in N1 were higher than in other treatments, while the Simpson index in N1 was lower than that in other treatments. Meanwhile, the N addition caused significant changes in the bacterial community structure (PERMANOVA test, *F* = 2.68, *p =* 0.001; Figure 3B). Further qPCR analysis showed that the activities of nitrogen-fixing microorganisms (NIFH), ammonia-oxidizing archaea (AOA), and ammonia-oxidizing bacteria (AOB) were stimulated with increasing N input, whereas the abundance of NIFH, AOA, and AOB showed the changing trend of first increasing, followed by the decrease, and then again the increase with the increase in the amount of added urea. The results indicated that lower N input may be beneficial to soil microbial diversity and species richness, especially nitrogen-fixing microorganisms (NIFH), ammonia-oxidizing archaea (AOA), and ammonia-oxidizing bacteria (AOB).

#### 3.3.2. Effects of N supply levels on the composition of soil bacterial community

**Figure 4:**
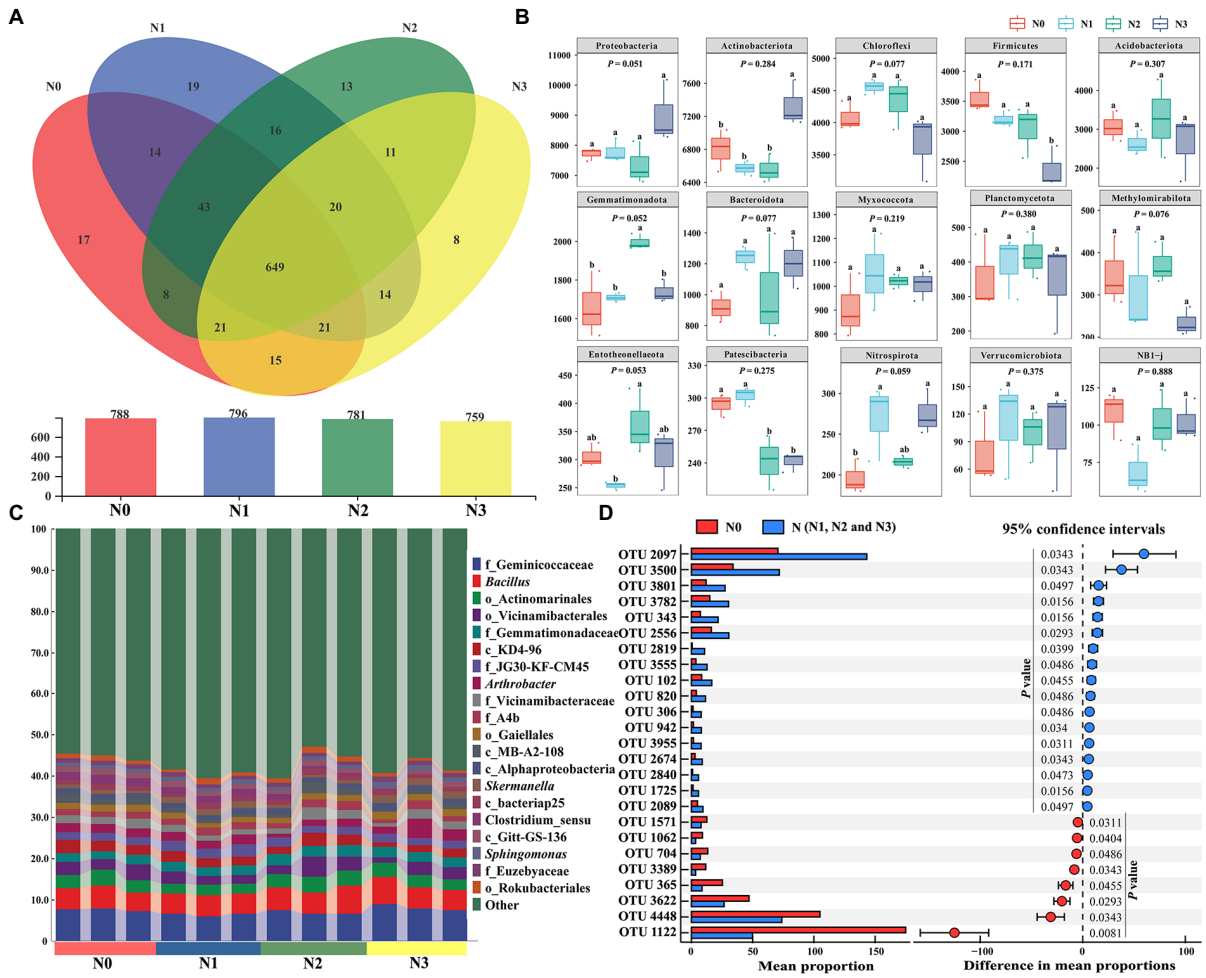
N-induced changes in bacterial community structure and biomarkers.

The Venn diagram drawn to reveal the OTUs at the genus level in different samples from different treatments showed that there were 788, 796, 781, and 759 bacterial genera in N0, N1, N2, and N3, respectively (Figure 4A). The number of bacterial genera shared between N0 and N (N1, N2, and N3) samples reached 771, including 17 unique for N0 and 101 unique for N (N1, N2, and N3). The N addition caused significant changes in bacterial community structure (Figures 3, 4A). *Proteobacteria*, *Actinobacteriota*, *Chloroflexi*, *Firmicutes*, *Acidobacteriota*, *Gemmatimonadota*, *Bacteroidota*, and *Nitrospirota* were the dominant phyla in soil bacterial community (Figure 4B). Meanwhile, there were significant differences in the relative abundance of *Actinobacteriota*, *Firmicutes, Gemmatimonadota, Entotheonellaeota, Patescibacteria*, and *Nitrospirota* among the groups N0, N1, N2, and N3. The relative abundance of *Nitrospirota* showed a significant increase in N (N1 and N3) samples when compared to N0, higher in N2 samples than in the N0 sample. The analysis of dominant bacteria at the genus level showed that 20 genera, including *Bacillus*, *Arthrobacter*, *Skermanella*, and *Sphingomonas*, were the dominant soil bacteria, accounting for 40–47% of the total bacterial abundance (Figure 4C). To compare the significantly different OTUs of bacteria in samples with and without the N input, we used the STAMP software to investigate the responses of bacterial OTUs to N input (Figure 4D). The N input significantly (*p* < 0.05) affected around 25 bacterial OTUs (Figure 4D and Supplementary Figure S3). The relative abundances of 17 bacterial OTUs, such as OTU2097, OTU3500, OTU3801, OTU3782, and OTU343, belonging to the phyla *Proteobacteria, Actinobacteriota, Chloroflexi, Bacteroidota, Gemmatimonadota, Acidobacteriota, RCP2-54*, and *Myxococcota*, increased significantly in N (N1, N2, and N3) samples compared to N0. The relative abundance of OTU1122 belonging to the *Terrisporobacter* genus of the phylum *Firmicutes* exhibited a significant decrease in N samples. In general, the N input changed the community structure and diversity of soil bacteria, such as *Nitrospirota*, *Bacillus*, *Streptomyces*, and *Sphingomonas*, which may have potential relevance to the cycling of soil nitrogen and other nutrients.

#### 3.3.3. Effects of N supply levels on N cycling

To compare the potential transformation mechanisms of nutrients in the rhizosphere soil of *L. barbarum* with and without N inputs, we used the Functional Annotation of Prokaryotic Taxa (FAPROTAX) to predict the ecological functions of rhizosphere soil bacteria (Figure 1A). The N input may have an influence on chemoheterotrophy, fermentation, nitrate reduction, ureolysis, chitinolysis, nitrogen fixation, cellulolysis, nitrate respiration, and other metabolic functions or pathways. Further PCoA analysis showed that the N addition caused significant changes in the function and structure of bacterial communities (PERMANOVA test, *F* = 16.127, *p* = 0.001; Figure 1B). The LEfSe analysis was conducted on soil nutrient cycling pathways to determine pathways at the FAPROTAX gene annotation with significant differences in abundance in different samples. Differences in persistent patterns of soil nutrient pathways between different levels of N input were revealed (Figure 1C). The 20 most important metabolites selected are mainly involved in anoxygenic photoautotrophy, aerobic chemoheterotrophy, chemoheterotrophy, chitinolysis, fermentation, nitrate reduction, cellulolysis, nitrification, etc. The N cycling pathways, such as aerobic chemoheterotrophy, nitrate reduction, nitrification, nitrite denitrification, ureolysis, nitrate respiration, nitrite ammonification, and nitrate denitrification, were different between the samples with and without N inputs. This indicated that N input had a significant impact on the nitrogen cycle in the rhizosphere soil of *L. barbarum*. Among nitrogen-cycling pathways, the activity of bacteria that have roles in aerobic chemoheterotrophy, nitrate reduction, nitrite denitrification, nitrate respiration, and nitrate denitrification increased with the increase in the N input level, whereas the activity of bacteria involved in nitrite ammonification decreased with the increase in N input level. Further STAMP analysis showed that the rates of nitrogen respiration, nitrite ammonification, and nitrite respiration significantly decreased in N1 samples compared to N0 (Figure 1D). The results of the redundancy analysis (RDA) showed that soil chemical properties, such as TN, NO_3_^−^, TK, pH, EC, OM, etc., could explain 26.17% of the total bacterial variation in microbial community structure, of which the first two explained 15.52 and 11.19% of the total variation, respectively (Supplementary Figures S4A,B). Meanwhile, the urea levels and soil chemical properties, such as NO_3_^−^, EC, TK, AP, and NH_4_^+^, could explain 65.51% of the total variation in biological functions or phenotype and 45.26% of the total variation in bacterial community functions, e.g., soil nitrogen cycling, of which the first two explained 24.72 and 20.54% of the variation in soil nitrogen cycling, respectively (Supplementary Figures S4C,D). Most soil chemical properties demonstrated significant relationships with both the bacterial alpha-diversity and nitrogen cycle when measured individually. The MANTEL analysis was performed to explain the relationships between the nitrogen cycle and soil chemical properties, bacterial alpha diversity, and markers to determine the bacterial populations involved in the N cycle under four treatments (Figure 1E). The results of the MANTEL test showed significantly positive correlations between urea levels and soil pH, EC, TN, TP, NO_3_^−^, AK, and AP and ureolysis, nitrate respiration, nitrite ammonification, nitrite respiration, and nitrate reduction. The abundance of nitrogen-fixing microorganisms (NIFH) and ammonia-oxidizing bacteria (AOB) was significantly positively correlated with aerobic nitrite oxidation, nitrification, and ureolysis.

#### 3.3.4. Intensity and structure of the functional genes involved in N cycling

**Figure 5:**
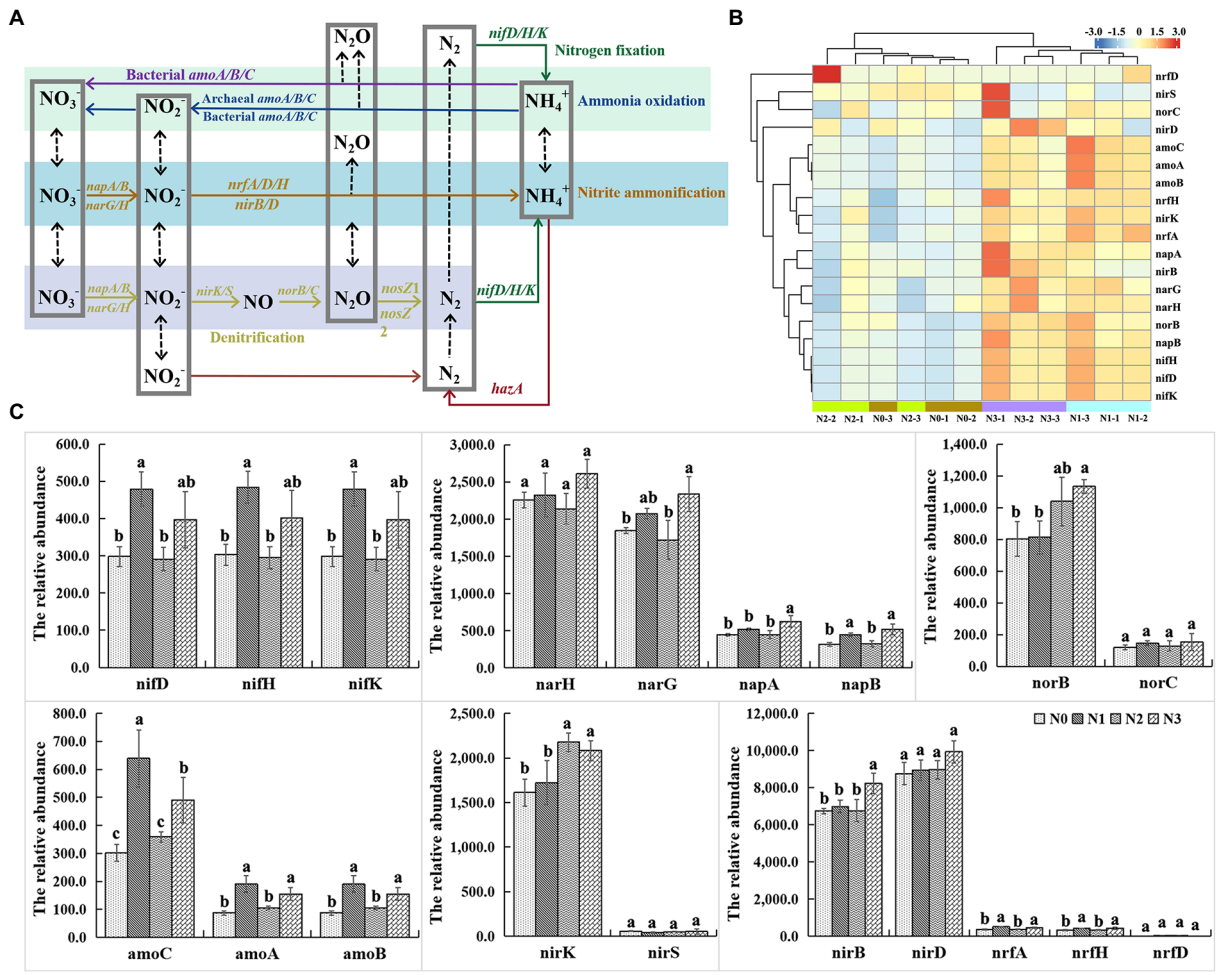
Influences of experimental N addition on nitrogen metabolism related genes.

A total of 19 functional genes were classified into 6 functional categories of N cycling in the rhizosphere soil of wolfberry in four N treatments (Figure 5). Among them, the nitrate reduction gene *narG*, nitrite reduction genes *nirS* and *nirK*, and N-fixation gene *nifH* were the top four functional genes involved in decomposition, denitrification, and N fixation, respectively. Besides these, denitrification genes *nrfA*, *napA*, and *napB*, and nitric oxide reductase genes *norB* and *norC*, were associated with microbial groups involved in dissimilatory nitrate reduction, ammonification, and denitrification. The change of specific genes involved in N cycling may indicate that the functional capacities of soil bacterial communities were affected by the amount of applied N. Heatmap displaying the results of cluster analysis showed that the signal intensity of N-cycling function genes was higher in N1 and N3 treatments compared with N0 and N2, and the gene expression patterns in the treatment groups of N0/N2 and N1/N3 were similar (Figure 5B). Results of the student–newman–keuls test showed significant changes in the signal intensity of *nifD*/*H*/*K*, *narG*/*H*, *napB*, and *amoA/B/C* genes under different treatments (Figure 5C). The signal intensity of *nifD*/*H*/*K* genes in different samples was in the order of N1 > N3 > N0 > N2, indicating that the microbial functional potentials of nitrogen fixation differed between different urea application levels. In addition, 19 functional N-cycling genes showed negative correlations with the content of NH_4_^+^ but positive correlations with the content of NO_3_^−^ and abundances of NIFH, AOA, and AOB (Supplementary Figure S5). However, it is strange that why the intensity of these genes in conventional N input treatment was lower than the reduced amounts of N input treatment and the increased amounts of N input treatment. We think it may be due to the fact that the nitrogen-fixing bacteria of the rhizosphere soil of wolfberry have adapted to the conventional applied urea level, and any increase or decrease would stimulate the activity of nitrogen-fixing bacteria.

#### 3.3.5. Effects of various bacterial parameters on the soil nitrogen cycle and nutrient contents of *Lycium barbarum* fruits

**Figure 6:**
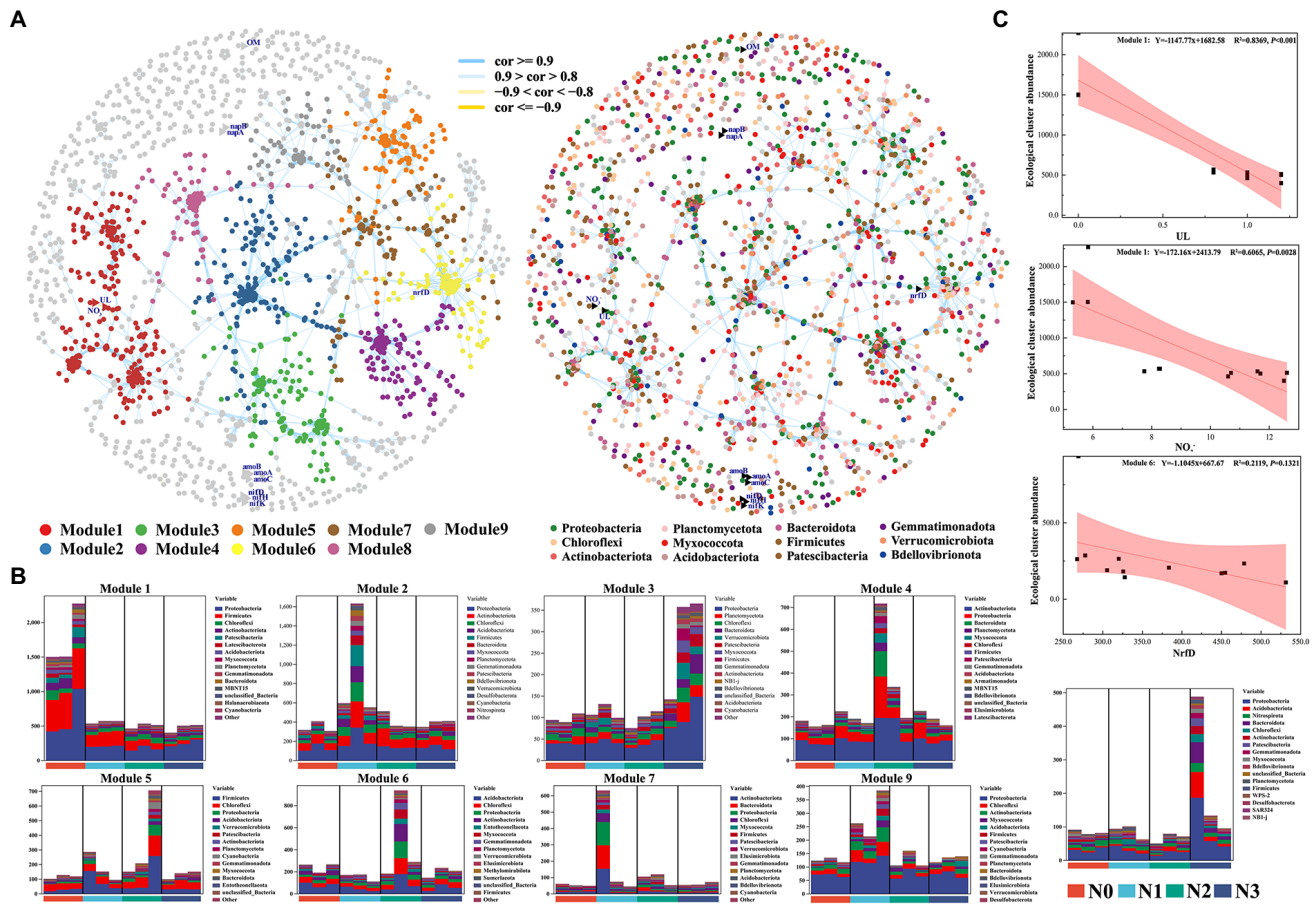
Response of co-occurrence networks of soil microbial communities to experimental N addition.

To investigate how bacterial parameters influence the soil nitrogen cycle and nutrient contents of wolfberry fruits, we constructed the co-occurrence networks of soil samples with (N1, N2, and N3) and without (N0) nitrogen inputs (Figure 6A). The node degrees of networks, the power-law degree distributions, and the average path length were significantly greater than those of the corresponding random networks, suggesting the co-occurrence networks to be the small-world networks. The co-occurrence network had 1822 nodes, including 1810 bacterial nodes and 12 Ul, OM, NO_3_^−^, *nrfD*, *nifK*, *nifH*, *nifD*, *napB*, *napA*, *amoC*, *amoB*, and *amoA* nodes, 40,413 edges, 38 phyla, and 42.23–54.78% of bacterial biomass. Of these nodes, 30.72, 17.48, 11.93, 9.86, 8.91, and 0.83% belonged to the phyla *Proteobacteria, Firmicutes, Actinobacteriota, Acidobacteriota, Chloroflexi,* and *Nitrospirota*, respectively, suggesting that the nodes of *Proteobacteria*, *Firmicutes* and *Actinobacteria* were more likely located at core positions in the network compared to other species (Figure 6B). To better examine the correlations between environmental factors, N cycle, and soil bacteria, the co-occurrence networks of bacterial OTUs from genes associated with environmental factors and nitrogen cycle were constructed. The results of the co-occurrence network analysis showed that UL, OM, and NO_3_^−^ and nine nitrogen-cycling genes were related to soil bacteria. Among them, UL and NO_3_^−^ had significant negative correlations with the bacteria from Module 1, and their relative abundances were higher in the N0 sample than in the N samples (Figure 6C). Meanwhile, some non-dominant bacteria involved in the soil nitrogen cycle, such as *nitrospira*, *pseudomonas*, *nitrosospira*, *bradyrhizobium*, *nitrosomonas*, *nitrolancea*, and *nitrococcus*, were from these nodes, among which, the relative abundance of *nitrospira, nitrosomonas,* and *nitrococcus* showed a strong linear relationship with the urea input levels (Supplementary Figure S6).

The Fla., Vit, TN, NO_3_^−^, and NH_4_^+^ were significantly (*p* < 0.05) influenced by UL, AOB, *bradyrhizobium*, and their interactions (Table 2). The results of linear regression showed that the content of NO_3_^−^ in the rhizosphere soil of wolfberry was significantly positively correlated with the urea application level; however, the contents of NO_3_^−^ and NH_4_^+^ in the rhizosphere soil were negatively correlated with the abundance of ammonia-oxidizing bacteria (AOB). Moreover, the content of TN in the rhizosphere soil and also the contents of flavones and vitamins in wolfberry fruits were positively correlated with the relative abundance of *bradyrhizobium* (Supplementary Figure S7).

**Table 2:** Effect of various ecological variables on the nutrient content of fruit and soil nitrogen content as estimated by GLMM showing significant effect on the final best fitting models.

Fixed effect factor	Dependent variable	Estimate	Std. Error	*t* value	*p* (>|*t*|)
(Intercept)	AA	0.7781	0.391	1.9902	0.1174
	Alk	0.0879	0.3573	0.246	0.8286
	Fla	−0.9607	0.3285	−2.9245	0.0769
	Sac	−0.2929	0.6921	−0.4232	0.7156
	Vit	0.1844	0.9751	0.1891	0.8759
	TN	0.2751	0.2219	1.2398	0.3440
	NO_3_^−^	0.0749	0.095	0.7888	0.4944
	NH_4_^+^	−0.2144	0.1779	−1.2052	0.3420
UL	AA	0.1504	0.8032	0.1873	0.8605
	Alk	0.3461	0.7303	0.474	0.6602
	Fla	1.2513	0.6267	1.9965	0.117
	Sac	−0.4851	0.8617	−0.563	0.6122
	Vit	−0.1867	1.0442	−0.1788	0.8801
	TN	0.3661	0.2549	1.4364	0.2593
	NO_3_^−^	0.7347	0.1671	4.3959	0.0118*
	NH_4_^+^	0.2146	0.2652	0.8093	0.4648
AOB	AA	0.272	0.4024	0.676	0.5361
	Alk	0.1578	0.3658	0.4312	0.6886
	Fla	0.5622	0.3173	1.7719	0.1532
	Sac	−0.0813	0.5363	−0.1516	0.8885
	Vit	−0.3673	0.41	−0.8958	0.4456
	TN	−0.1816	0.1489	−1.2195	0.2914
	NO_3_^−^	−0.2907	0.0876	−3.3188	0.0351*
	NH_4_^+^	−0.5397	0.1542	−3.4998	0.0422*
*Bradyrhizobium*	AA	0.8253	0.8565	0.9636	0.3898
	Alk	0.9049	0.7792	1.1613	0.3107
	Fla	−2.3155	0.6696	−3.4581	0.0266*
	Sac	1.4928	0.6762	2.2076	0.1029
	Vit	1.6769	0.3761	4.4585	0.0495*
	TN	0.4904	0.1605	3.0554	0.0487*
	NO_3_^−^	0.2508	0.1761	1.4246	0.2327
	NH_4_^+^	−0.3711	0.2574	−1.4418	0.2327
UL: AOB	AA	0.6649	1.4488	0.4589	0.6701
	Alk	0.944	1.3131	0.7189	0.5253
	Fla	−1.3338	1.081	−1.2339	0.3068
	Sac	1.0506	0.9542	1.101	0.3522
	Vit	0.1832	0.4839	0.3786	0.7488
	TN	−1.0255	0.2181	−4.701	0.0205*
	NO_3_^−^	−0.1107	0.2741	−0.4041	0.7130
	NH_4_^+^	−0.0211	0.3822	−0.0553	0.9594
UL: *Bradyrhizobium*	AA	−1.9754	1.6081	−1.2284	0.2866
	Alk	−3.9046	1.461	−2.6726	0.0569
	Fla	5.9877	1.2364	4.8428	0.0105*
	Sac	−1.7825	1.1404	−1.5631	0.2184
	Vit	−2.0385	0.569	−3.5824	0.0988
	TN	−0.1176	0.2594	−0.4535	0.6833
	NO_3_^−^	−0.2363	0.3204	−0.7374	0.5081
	NH_4_^+^	1.5216	0.4557	3.3388	0.0415*
AOB: *Bradyrhizobium*	AA	0.9153	1.034	0.8852	0.4261
	Alk	−0.4311	0.9383	−0.4595	0.6727
	Fla	−1.102	0.786	−1.402	0.2429
	Sac	−0.3123	0.8467	−0.3688	0.7313
	Vit	0.7879	0.5317	1.4818	0.2672
	TN	1.1258	0.212	5.3107	0.0069**
	NO_3_^−^	0.1032	0.2038	0.5061	0.6422
	NH_4_^+^	0.601	0.302	1.9903	0.1209
UL: AOB: *Bradyrhizobium*	AA	−1.5065	2.364	−0.6373	0.5586
	Alk	−5.8477	2.1449	−2.7263	0.0616
	Fla	7.5149	1.7891	4.2003	0.0203*
	Sac	−3.2334	1.6318	−1.9814	0.1408
	Vit	−3.0302	0.8383	−3.6148	0.0909
	TN	−0.0675	0.375	−0.18	0.869
	NO_3_^−^	−0.2673	0.4587	−0.5828	0.598
	NH_4_^+^	1.8745	0.6477	2.894	0.0595

UL, the level of nitrogen (urea) input. * indicates a significant influence at the 0.05 level, ** indicates a significant influence at the 0.01 level.

## 4. Discussion

The soil contains a diverse community of microorganisms, primarily bacteria, fungi, archaea, protozoa, and viruses, which play an important role in soil nitrogen supply and crop nitrogen absorption by driving the biogeochemical nitrogen cycle and plant nitrogen acquisition in agricultural ecosystems (Canfield et al., 2010; Philippot et al., 2013). Plants absorb nitrogen in three forms: ammonium, nitrate, and organic nitrogen (Peng et al., 2022). Soil microorganisms play an irreplaceable role in the process of nitrogen assimilation into ammonium and nitrate and are widely involved in six distinct nitrogen transformation processes, such as nitrogen fixation, in which dinitrogen gas is first fixed to ammonia by plants and microorganisms, ammonification, in which organic nitrogen degradation releases ammonium, nitrification, in which NH_4_^+^ is oxidized to nitrate (NH_4_^+^ → NO_2_^−^ → NO_3_^−^), denitrification (NO_3_^−^ → NO_2_^−^ → NO→N_2_O → N_2_) or anaerobic ammonium oxidation (NH_4_^+^ + NO_2_^−^ → N_2_; Peng et al., 2022). Soil bacteria have more nitrogen metabolism genes than archaea and fungi, and they are versatile in nitrogen metabolism, being involved in all stages of the nitrogen cycle (Cabello et al., 2004; Li et al., 2020).

The rhizosphere is the most intricately connected microenvironment of plants, soil, and microbes. Plant roots obtain nitrogen directly from the rhizosphere soil-root region in order to maintain growth and development, and nitrogen deficiency has a direct impact on crop growth, yield, and quality. Urea is one of the most widely used nitrogen fertilizers in agricultural production, and it is critical to maintaining global food production and advancing modern agriculture. Urea fertilizers are rapidly converted to ammonium by microbial action in soils. After applying urea to the soil, the activity of soil urease can be increased, allowing it to hydrolyze urea and increase NH_4_^+^-N content, promoting nitrification to form NO_3_^−^-N (Ma et al., 2007; Wang et al., 2020). Urease is an enzyme found in many soil bacteria that catalyzes the conversion of urea to ammonia or ammonium ions and bicarbonate ions. Some ammonia-oxidizing bacteria (AOB), such as *Nitrosomonas* species, can assimilate the carbon dioxide released by the reaction to make biomass *via* the Calvin Cycle and harvest energy by oxidizing ammonia (the other product of urease) to nitrite, a process known as nitrification (Marsh et al., 2005). The AOB can convert the most reduced form of N (ammonia) into nitrate, and this is the first step in the nitrification process, which is very important. In our study, after adding urea to the soil, the relative abundance of AOB and the activity of urease in the rhizosphere soil of *L. barbarum* increased significantly, but as urea use increased, the abundance of AOB and the activity of urease decreased significantly. Meanwhile, the amount of soil nitrogen input influences soil bacterial diversity and community structure, and excessive fertilization reduces alpha diversity of bacteria, and the contents of flavonoids, alkaloids, and amino acids in *L. barbarum* fruits were found to be negatively correlated with urea levels. However, nitrogen deficiency stress (N0) significantly inhibited the biosynthesis of nutrients in *L. barbarum* fruit, such as flavonoids, alkaloids, vitamins, and amino acids. This is also consistent with previous research findings (Shen et al., 2010). Under different nitrogen supply conditions, the structure and composition of the bacterial community in wheat roots were significantly different, and the addition of high nitrogen would inhibit the diversity and stability of the bacterial community (Kavamura et al., 2018). Under low nitrogen conditions, the Shannon diversity index of the plant rhizosphere soil bacterial community is higher than under high nitrogen conditions (Shen et al., 2010). *Proteobacteria* and other eutrophic bacteria thrive in environments with abundant nitrogen (Zeng et al., 2016). The competitive advantage of more oligotrophic microorganisms, such as *Chloroflexi*, *Firmicutes*, *Bacteroidota*, and *Patescibacteria*, over eutrophic microorganisms under nitrogen deficiency stress may lead to greater soil microbial diversity.

Fertilization, along with temperature and precipitation, has a greater impact on the soil nitrogen cycle in the farmland ecosystem. Different fertilization methods, particularly the combination of chemical and organic fertilizer, have different effects on the structure and abundance of functional genes related to the soil nitrogen cycle. Meanwhile, each process of the soil nitrogen cycle is interconnected, and it may be impossible to evaluate the impact of fertilization mode on it comprehensively by studying a single nitrogen cycle gene singly. Different nitrogen cycle processes have their own functional genes that play a key role, such as *nifH* in N_2_ fixation, *amoA/B* in nitrification, *narG*, *napA*, *nirK/S*, and *nosZ* in denitrification, and *nirB/D* and *nrfA/H* in nitrate dissimilatory reduction (He et al., 2022; Li et al., 2020). According to our findings, the urea added amount has a significant impact on nitrogen cycle-related processes such as ureolysis, aerobic nitrite oxidation, nitrate denitrification, nitrate reduction, nitrate respiration, nitrification, nitrite ammonification, nitrogen fixation, nitrogen respiration, and nitrous oxide denitrification. Further analysis of soil nitrogen cycle genes revealed that reducing or increasing nitrogen fertilizer (N1 and N3) application would affect soil nitrogen fixation (*nifD/H/K*), nitrification (*amoA/B/C*), and nitrite ammonification (*narG/H*, *napB*, *nirB*, and *nrfA/H*) compared to normal urea application (N2). The signal intensity of *narG*/*H* and *napA*/*B* genes in the conventional N input (N2) treatment was lower than that in treatments receiving the reduced amounts of N input (N1) and the increased amounts of N input (N3), indicating that functional potentials of the denitrification process, in which nitrate is reduced to nitrite, differed between N2 and N1/N3 application levels. The signal intensity of *amoA*/*B/C* genes in the conventional N input (N2) treatment was lower than that in treatments receiving reduced amounts of N input (N1) and increased amounts of N input (N3), indicating the enhanced functional potentials of ammonia oxidation in N1 and N3 samples. On average, the expression levels of all N-cycling functional genes from all four treatments substantially increased at the reduced N application level (N1), and the increased level of N input (N3) compared to those in the conventional N application (N2) level. In traditional agriculture, the nitrogen fixation of plant rhizosphere bacteria is the most important source of nitrogenous fertilizer. Higher chemical N fertilizer inputs, on the other hand, can reduce the agroecosystem’s reliance on free-living N-fixing bacteria, stimulate resource competition, and further inhibit N fixation (Yu et al., 2021). Ammonia oxidizing bacteria (AOB) are critical nodes in the microbial coexistence network and an important participant in the soil nitrogen cycle, catalyzing the first step in the ammonia oxidation process (Francis et al., 2005; Gao et al., 2013). *Nitrosospira* is an important species of ammonia oxidizing bacteria, and low nitrogen input stimulates *Nitrosospira* relative abundance increasing, it indicate that reducing nitrogen fertilizer use may be more beneficial to the soil nitrogen cycle.

The modern multiomics technology, such as macromicrobiology, macrotranscriptomics, and metabolomics, have increased our understanding of plant microbiomes and their roles in coordinating soil nitrogen supply and crop nitrogen absorption, as well as revealed the possibility of manipulating microbiomes to improve crop nitrogen utilization efficiency and reduce fertilizer use. The GLMM model results show that the amount of nitrogen (urea) input, the relative abundance of AOB, and the relative abundance of *Bradyrhizobium* all have a significant effect on the content of NO_3_^−^ and NH_4_^+^ in soil, as well as the quality of wolfberry fruit. Reduced nitrogen fertilizer input has no effect on wolfberry fruit quality, and can supply plant nutrient by stimulating soil nitrogen fixation and the nitrogen cycle of soil bacteria. This research lays the foundation for future research into the physiological and ecological functions of nitrogen-transforming microorganisms and their evolution, as well as the regulation mechanism of *L. barbarum* rhizosphere nitrogen utilization. It is critical for understanding the mechanisms of nitrogen absorption and transport in wolfberry roots and improving wolfberry nitrogen use efficiency. It is an important reference for precise fertilization, nitrogen savings, and efficiency improvement in the production of *L. barbarum*.

## 5. Conclusion

We discovered that changing the nitrogen input can affect the quality of *L. barbarum* fruit by altering the soil microbial community structure and the soil nitrogen cycle in the rhizosphere. The GLMM model results show that the amount of nitrogen (urea) input, the relative abundance of AOB, and the relative abundance of *Bradyrhizobium* all have a significant effect on the content of TN, NO_3_^−^ and NH_4_^+^ in the soil as well as the biosynthesis of nutrients in *L. barbarum* fruit. As a result, we believe that moderately reducing the use of urea and other nitrogen fertilizers is more conducive to improving soil nitrogen use efficiency and goji berry quality.

## Data availability statement

The original contributions presented in the study are publicly available. This data can be found here: https://www.ncbi.nlm.nih.gov/; accession number PRJNA904642.

## Author contributions

YL and JL developed the concept of this study and are main contributors to writing the manuscript. YT, NZ, XL, and XZ performed all experiments, data analysis, and prepared figures. SG, YW, XQ, and JL contributed to the manuscript edit and review. All authors contributed to the article and approved the submitted version.

## Funding

This work was supported by Grants-in-Aid for scientific research from the Natural Science Foundation of Ningxia (2021AAC03261) and National Natural Science Foundation of China (31860150).

## Conflict of interest

The authors declare that the research was conducted in the absence of any commercial or financial relationships that could be construed as a potential conflict of interest.

## Publisher’s note

All claims expressed in this article are solely those of the authors and do not necessarily represent those of their affiliated organizations, or those of the publisher, the editors and the reviewers. Any product that may be evaluated in this article, or claim that may be made by its manufacturer, is not guaranteed or endorsed by the publisher.

## Footnotes

^1^ https://docs.qiime2.org/2019.4/tutorials/
