# Supplementary material for: Effects of nitrogen input on soil bacterial community structure and soil nitrogen cycling in the rhizosphere soil of Lycium barbarum L
Source: Front Microbiol. 2023 Jan 10;13:1070817. doi: 10.3389/fmicb.2022.1070817 (PMC9871820; doi:10.3389/fmicb.2022.1070817)
Supplement: Supplementary file 1 [file Data_Sheet_1.docx]

Table S 1 Annual fertilization amount of 0.0667 hectares of *Lycium barbarum* for each treatment

| ID | Treatment | Urea（kg/0.0667 hectares） | Overbearing Calcium Phosphate（kg/0.0667 hectares） | Potassium sulfat（kg/0.0667 hectares） |
| --- | --- | --- | --- | --- |
| N0 | Blank | 0 | 65.2 | 40 |
| N1 | Nitrogen reduction 20% | 78 | 65.2 | 40 |
| N2 | Conventional nitrogen | 98 | 65.2 | 40 |
| N3 | Nitrogen increase reduction 20% | 117 | 65.2 | 40 |

Table S2 The q-PCR primers of NIFH, AOA, AOB

| Gene | Primer | Sequence | Product size (bp) | Reference |
| --- | --- | --- | --- | --- |
| *NifH* | *nifH*-F | AAAGGYGGWATCGGYAARTCCACCAC | 450 | Francis et al., 2005 |
|  | *nifH*-R | TTGTTSGCSGCRTACATSGCCATCAT |  |  |
| *AOB* | *AmoA*-1F | GGGGTTTCTACTGGTGGT | 491 | Mao et al., 2011 |
|  | *AmoA*-2R | CCCCTCKGSAAAGCCTTCTTC |  |  |
| *AOA* | *Arch-amoA*F | STAATGGTCTGGCTTAGACG | 635 | Fan et al., 2019 |
|  | *Arch-amoA*R | GCGGCCATCCATCTGTATGT |  |  |


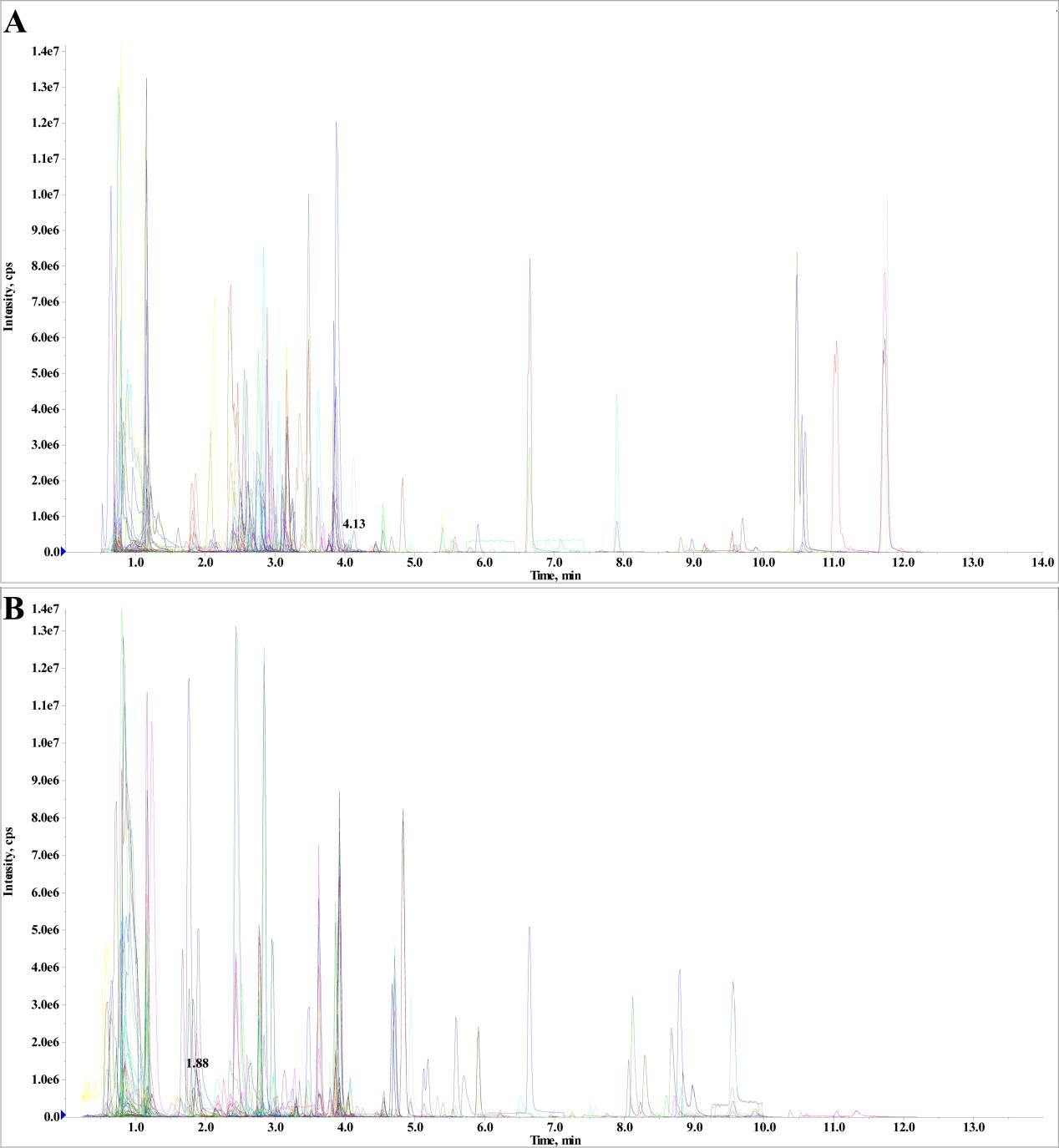


Figure S1 Total iron current chromatogram of a quality control (QC) samples by MS under the multiple reaction monitoring (MRM) mode (A and B for negative mode and positive mode, respectively).

Table S3 Correlation between soil enzyme activity and N addition and N content based on Spearman

|  | NAG (nmol/g/h) | LAP (nmol/g/h) | UR (ug/g/h) |
| --- | --- | --- | --- |
| UL | -0.086 | 0.238 | -0.194 |
| TN | 0.500 | 0.169 | 0.972** |
| NO_3_^-^ | -0.098 | 0.287 | -0.189 |
| NH_4_^+^ | 0.259 | 0.357 | -0.573 |


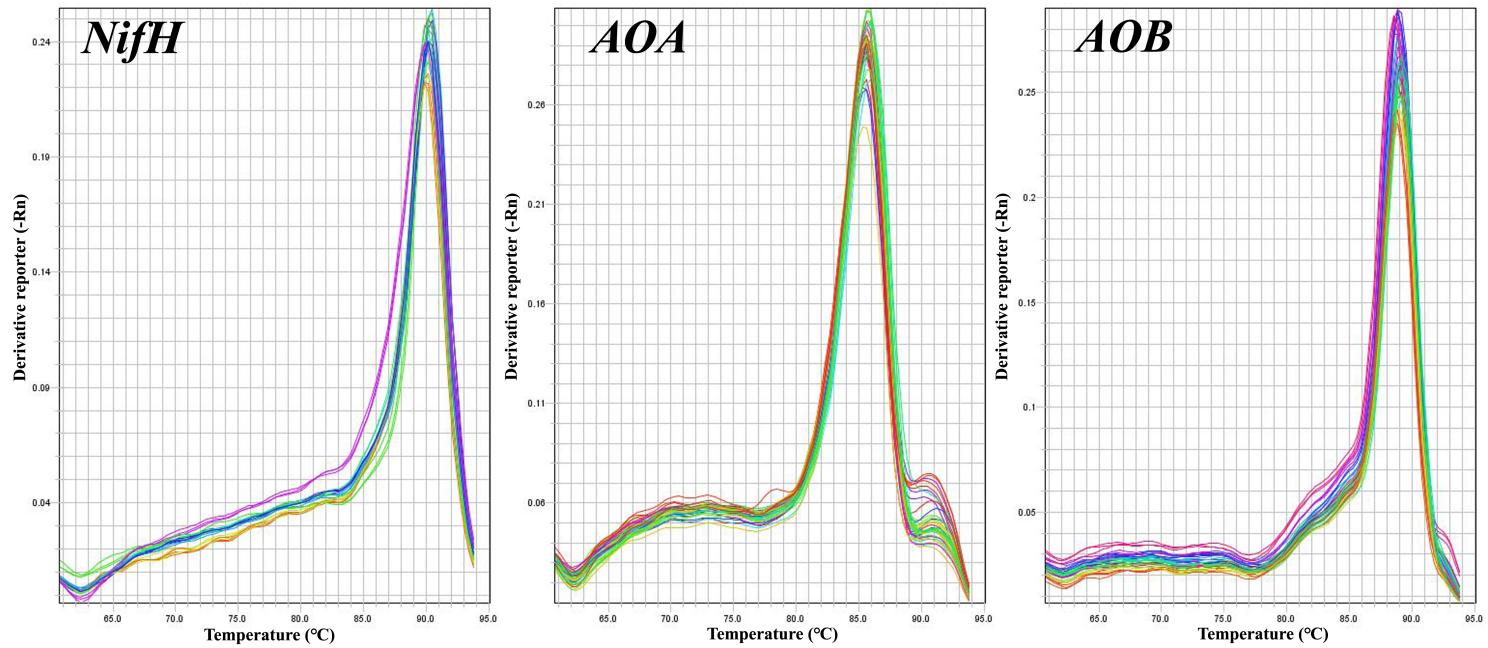


Figure S2 The melting curve of primer *nifH*-F/*nifH*-R, *Arch-amoA*F/*Arch-amoA*R, and *AmoA*-1F/*AmoA*-2R


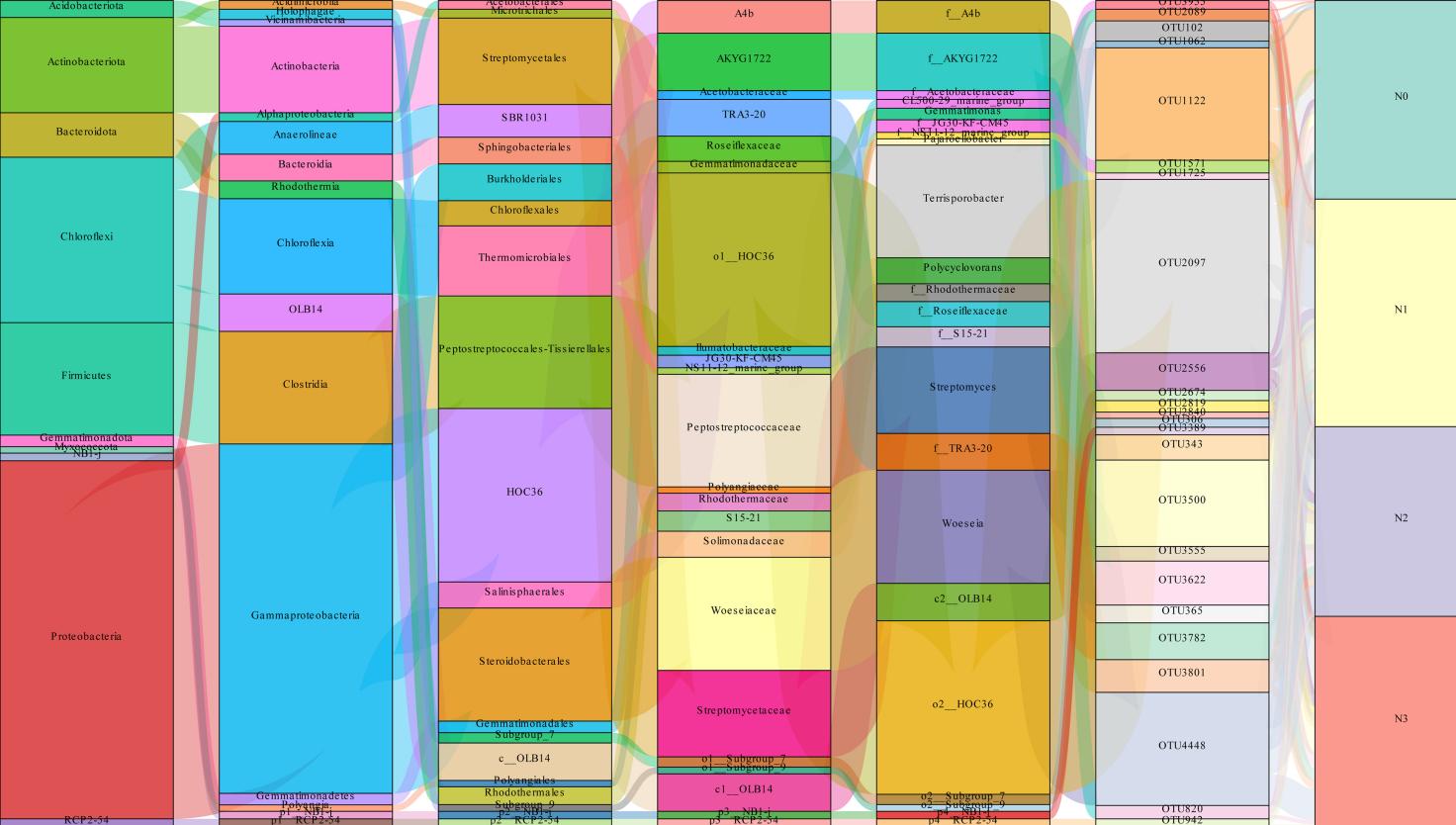


Figure S3 The corresponding relationship of OTUs in Figure 3D between different levels and the corresponding abundance information based on Sankey diagram


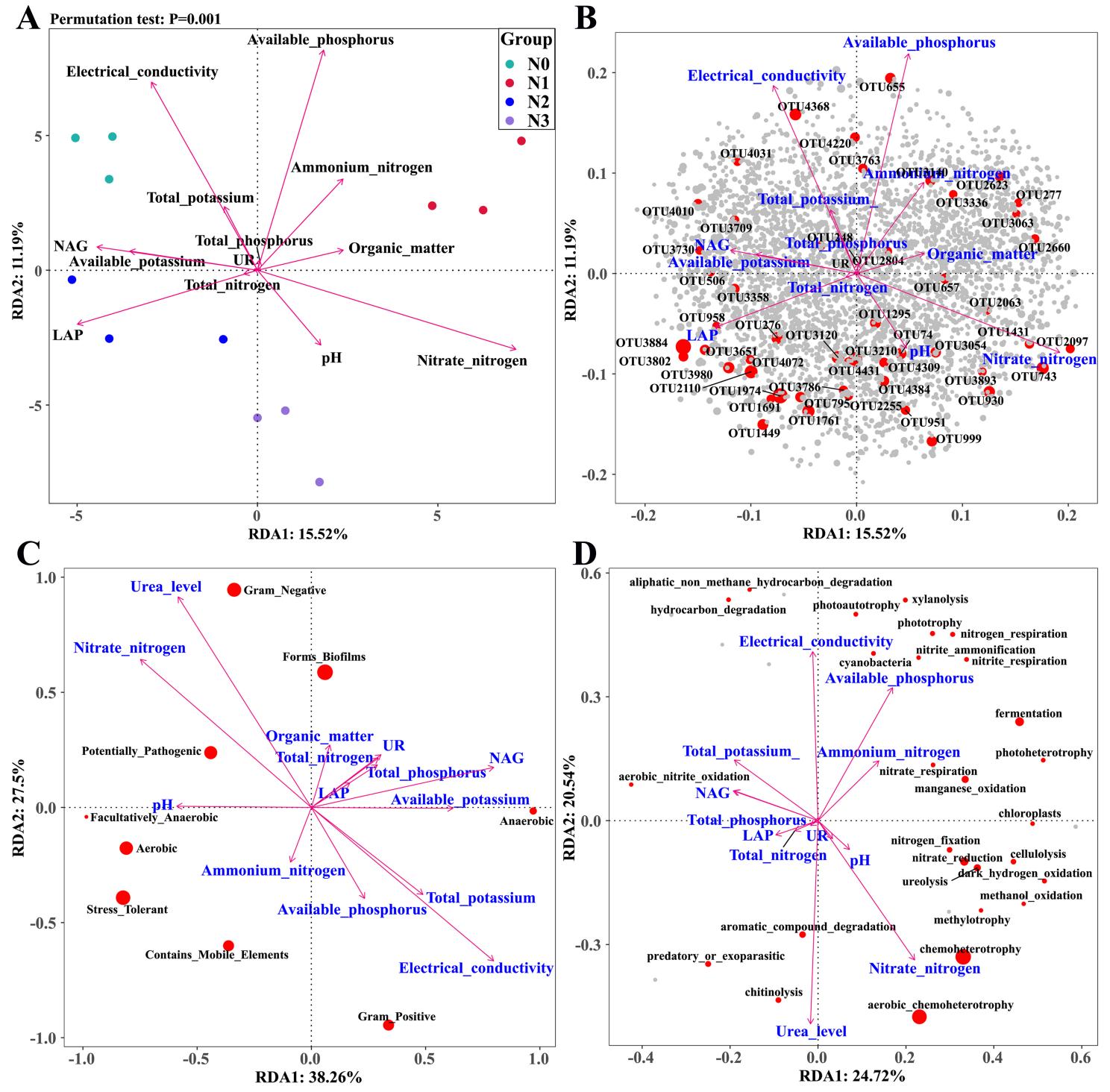


Figure S4 Redundancy analysis (RDA) of sequence data from 12 rhizosphere soil samples in *L. barbarum* with soil chemical properties and soil enzyme activities for soil bacterial structure (A and B) in wolfberry rhizosphere, bacterial biological function or phenotype (C), and functional gene of nitrogen cycle (D) associated with bacterial community structure.


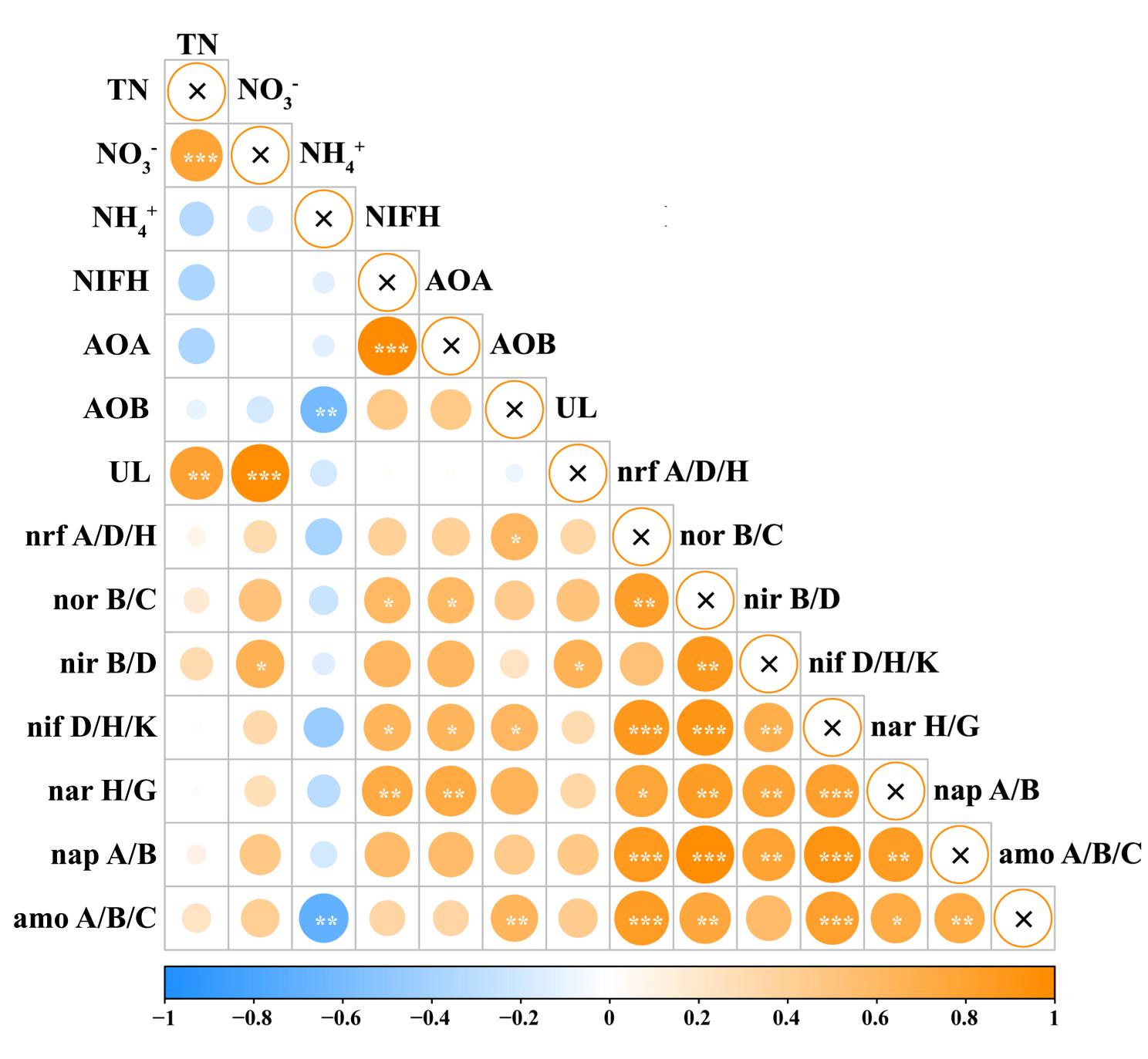
Figure S5 The spearman relationships between urea level, the relative abundance of AOA, AOB and the genes related to soil N cycling.


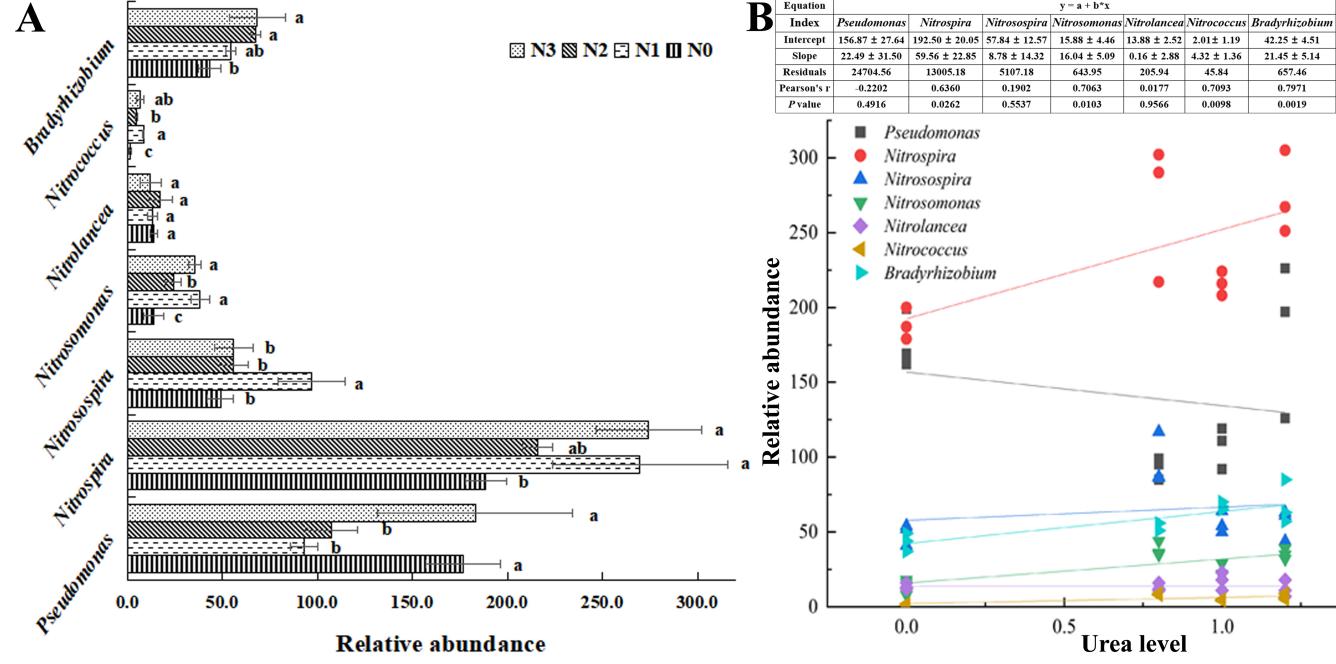


Figure S6 The linear relationships between urea level and major bacterial genera related to soil N cycle


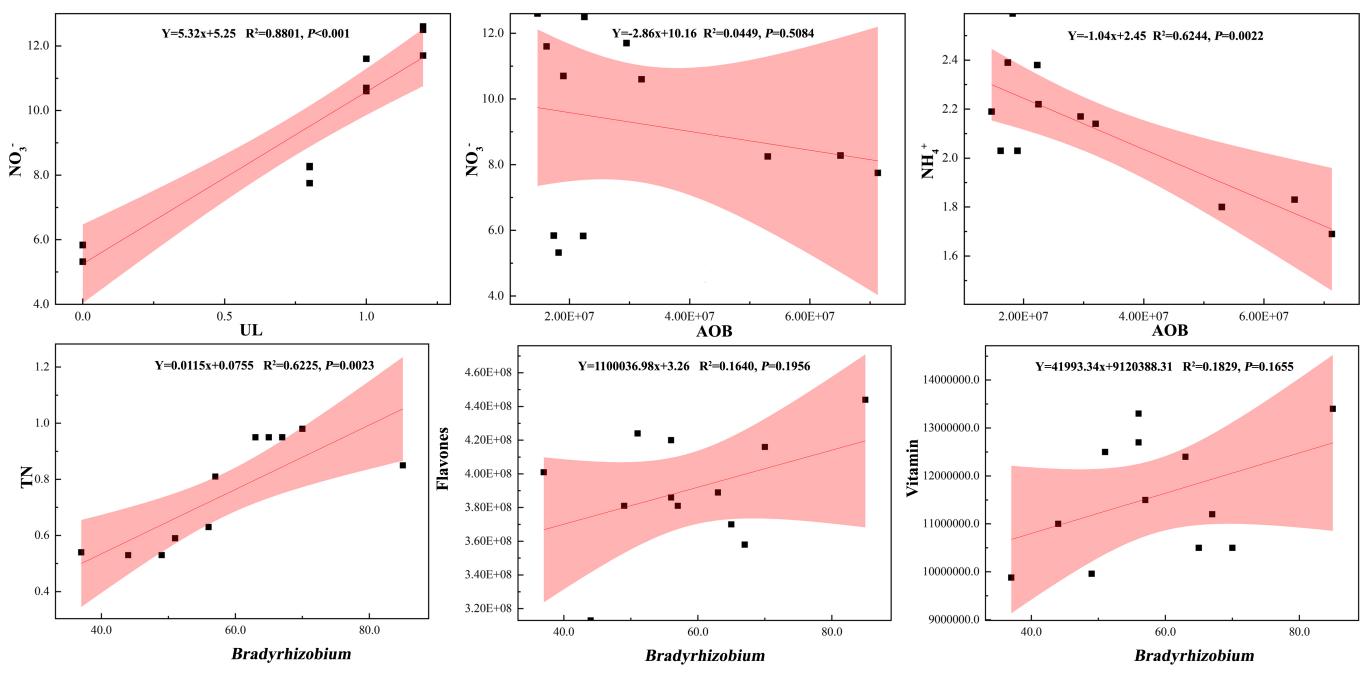


Figure S7 The linear relationships between urea level, the relative abundance of AOA, AOB and *Bradyrhizobium* and soil N, the major DEMs relative content of *Lycium chinensis* fruit.
